# IL-17A in Human Respiratory Diseases: Innate or Adaptive Immunity? Clinical Implications

**DOI:** 10.1155/2013/840315

**Published:** 2013-01-17

**Authors:** Dominique M. A. Bullens, Ann Decraene, Sven Seys, Lieven J. Dupont

**Affiliations:** ^1^Pediatrics Division, University Hospitals Leuven, 3000 Leuven, Belgium; ^2^Laboratory of Pediatric Immunology, Department of Microbiology and Immunology, KU Leuven, 3000 Leuven, Belgium; ^3^Laboratory of Experimental Immunology, KU Leuven, 3000 Leuven, Belgium; ^4^Laboratory of Pneumology, Department of Clinical and Experimental Medicine, KU Leuven, 3000 Leuven, Belgium; ^5^Pneumology Division, University Hospitals Leuven, 3000 Leuven, Belgium

## Abstract

Since the discovery of IL-17 in 1995 as a T-cell cytokine, inducing IL-6 and IL-8 production by fibroblasts, and the report of a separate T-cell lineage producing IL-17(A), called Th17 cells, in 2005, the role of IL-17 has been studied in several inflammatory diseases. By inducing IL-8 production and subsequent neutrophil attraction towards the site of inflammation, IL-17A can link adaptive and innate immune responses. More specifically, its role in respiratory diseases has intensively been investigated. We here review its role in human respiratory diseases and try to unravel the question whether IL-17A only provides a link between the adaptive and innate respiratory immunity or whether this cytokine might also be locally produced by innate immune cells. We furthermore briefly discuss the possibility to reduce local IL-17A production as a treatment option for respiratory diseases.

## 1. Introduction

In 1995, Yao and coworkers discovered a cytokine, produced by human T cells, which induced IL-6 and IL-8 production and enhanced the surface expression of the intracellular adhesion molecule-1 (ICAM-1) by human fibroblasts [1]. Since then, several IL-17 family members have been described [2–4]. The family member, which was originally described, is referred to as IL-17A ever since. More recently, a separate T-cell lineage, called Th17 cells or inflammatory T cells, producing IL-17A, has been identified [5, 6]. Th17 cells might potentially play an important role in the pathophysiology of respiratory diseases such as asthma, chronic obstructive pulmonary disease (COPD), cystic fibrosis (CF), and lung transplant rejection. Human Th17 cells express retinoic acid receptor-related orphan receptor (ROR)C and produce, besides IL-17A, also IL-17F, IL-21, IL-22, and IL-26 [7, 8]. Human Th17 cells furthermore express CCR6 [8]. Patients with defective Th17 cells are known to suffer from severe infections by fungi and extracellular bacteria such as *Candida albicans *and *Staphylococcus aureus* [8]. Even Th17 cells might form a heterogeneous family: indeed, human hybrid Th17/Th1 cells (producing both IL-17A and IFN-*γ* as well as expressing both RORC and T-bet), human hybrid Th17/Treg cells (expressing CCR6, RORC and Foxp3), and human Th17/Tr1-like cells (producing both IL-17 and, for a limited period of 3 to 5 days after initial stimulation, also IL-10) have been described [8]. There recently has been a lot of interest in the local microbiota interacting with specific T-cell subsets, and hereby controlling recruitment and further differentiation of certain T-cell subsets. *Candida albicans*-specific Th17 cells are hybrids and produce IL-17 and IFN-*γ*, but no IL-10, whereas *Staphylococcus aureus*-specific Th17 cells produce IL-17 and also IL-10 upon restimulation [9]. IL-6, IL-23, and IL-1*β* were shown to contribute to Th17 differentiation induced by both pathogens. However, amongst these cytokines, IL-1*β* was essential in *C. albicans*-induced Th17 differentiation to counteract the inhibitory activity of IL-12 and to prime for IL-17/IFN-*γ* double-producing cells [9]. On the other hand, germination and biofilm formation of certain fungi can also be induced upon their interaction with “host” IL-17A [10]. *Aspergillus fumigatus* can increase its biofilm formation upon binding IL-17A and its filamentation can be promoted, inducing resistance to local antifungal defenses [10]. This interaction might be of utmost importance in CF patients, in whom the defective clearance of certain pathogens leads to chronic colonization and/or infection with certain pathogens, including *Staphylococcus aureus* and/or *Aspergillus fumigatus.*

It still remains enigmatic whether the cited respiratory diseases are specifically mediated by local Th17 cells, able to produce several other cytokines, or by the cytokine IL-17A itself, which might be secreted besides by Th17 cells, by several other cell types. Indeed, IL-17A can also be produced by *γδ*T cells, NKT cells, and innate lymphoid cells, such as NK cells, lymphoid tissue inducer (LTi) cells, and innate lymphoid cells with specific IL-17A-secreting capacity, ILC17 [11, 12]. In humans, LTi cells and ILC17 cells might be overlapping cell populations [11]. Moreover, in murine models, IL-17A production in the lungs during invasive fungal infection was even shown to be mediated by CD11b(+) Ly-6G(+) neutrophils [13] and by alveolar macrophages upon allergen challenge [14]. Human neutrophils from cystic fibrosis patients were also shown to produce IL-17A [15].

Human IL-17A binds to its receptor IL-17RA, which, albeit weakly, also binds IL-17F [12]. IL-17RA binds with IL-17RC and can form heterodimers for optimal signalling [12]. IL-17RA is highly expressed in haematopoietic tissues and main responses to IL-17A are observed in epithelial cells, endothelial cells, fibroblasts, macrophages, and dendritic cells [12]. Several airway cells might therefore respond locally to IL-17A.

 IL-17A is especially important for the recruitment of neutrophils [16]. The mechanism of neutrophil recruitment to the airways is still unclear, but several chemokines were suggested to play a role [17]. Among those, CXCL8 (IL-8), secreted by T lymphocytes, epithelial cells, smooth muscle cells, and macrophages might especially be important, as CXCL8 (IL-8) is also increased in the airways of patients with some respiratory diseases such as CF, COPD, and asthma [18–22]. Neutrophil recruitment is an utmost important defence mechanism against local pathogens, but persisted neutrophil recruitment might lead to chronic inflammation with local production of neutrophilic mediators, probably involved in the physiopathology of different respiratory diseases. 

IL-17 (and IL-6) are furthermore major inducers of mucin genes *in vitro*, suggesting that IL-17 production might also be responsible for mucus secretion in IL-17-mediated respiratory diseases [23]. We here review the role of IL-17A and neutrophil recruitment in several human respiratory diseases: asthma, COPD, CF, and lung transplant rejection.

## 2. IL-17A in Asthma

The role of IL-17A and/or Th17 cells in asthma has been extensively studied in mouse models (reviewed in [24]). However, human data are scarce. Studies to unravel the role of IL-17A in asthma started in the late nineties by expression studies in airway cells. IL-17A was shown to be expressed in bronchial biopsies, bronchoalveolar lavage fluid and sputum of patients with asthma [22, 25–27]. We were the first to show that compared to healthy control individuals, sputum IL-17A and IL-8 mRNA levels were significantly higher in mild and moderate-to-severe asthmatics, whereas sputum IL-17A and IL-8 mRNA expression was similarly distributed in nonallergic and allergic patients [22]. Later on Doe and coworkers observed increased numbers of IL-17A^+^ cells in bronchial biopsies of mild asthmatics compared to healthy controls, whereas IL-17F^+^ cells were increased in mild and especially in moderate-to-severe asthmatics [28]. A recent study furthermore showed that upon bronchial house-dust-mite challenge systemic IL-17A levels increase in house-dust-mite allergic individuals [29]. Although this increase was clearly demonstrated, it remains unknown whether IL-17 (or Th17 cells?) is (are) increased in an attempt to protect the individual, or in contrary, might cause local harm. IL-17A is thought to be responsible for neutrophil recruitment, as a result of chemokine induction, such as IL-8. Neutrophils are especially prominent in acute, severe exacerbations of asthma [30, 31]. The subgroup of patients with neutrophilic asthma is characterized by poor response to corticosteroids [32]. Neutrophils potentially contribute to airway gland hypersecretion, bronchial hyperreactivity and to airway wall remodelling [33, 34] by producing matrix metalloproteinase-9 (MMP-9) observed in bronchoalveolar lavage fluid from moderate-to-severe asthma patients [35]. We found in our asthmatic study population that both sputum IL-17A and IL-8 mRNA levels correlate significantly with the sputum neutrophil count [22]. This correlation was also observed by other authors at the protein level [26]. A significant correlation between IL-8 mRNA and IL-17A mRNA on the one hand and with neutrophils on the other hand suggests (although it does not provide proof for) cause-effect relationship. On the other hand, Doe and coworkers observed that the number of IL-17A^+^ cells was correlated with FEV_1_% predicted (*R*_*s*_ = 0.38;  *P* = .04), and the sputum neutrophil count (*R*_*s*_ = −0.43,*P* = .03) in asthma but was not associated with the number of neutrophils or eosinophils in the bronchial mucosa [28]. They furthermore reported a good correlation between the number of IL-17F^+^ cells and eosinophils in the bronchial submucosa [28]. Human bronchial epithelial cells have recently been shown to secrete IL-17F in response to IL-33 [36]. IL-33, secreted by epithelial cells or macrophages (innate “immune” cells), might therefore induce IL-17F production by epithelial cells, leading to eosinophil recruitment in initial asthma [37]. Later on IL-17A, produced by both innate and adaptive immune cells, can induce neutrophil recruitment mediated by IL-8 secretion, leading to persistent neutrophilic asthma.

## 3. IL-17A in COPD

COPD is characterized by airway obstruction as well as emphysematous destruction, secondary to chronic inflammation induced by cigarette smoke, involving neutrophil recruitment. 

As for asthma, human studies on the potential involvement of IL-17 in COPD, started with expression studies in sputum or BAL from COPD patients [27, 28]. However, levels of IL-17 in sputum of COPD patients were significantly lower than in asthma (*P* = 0.004) and did not differ from levels in healthy controls [27]. Even decreased serum and sputum IL-17A levels in COPD patients compared to healthy controls have been reported [38]. More recently Doe and coworkers, on the other hand, were able to demonstrate that smoking was associated with increased numbers of IL-17A^+^ cells in bronchial biopsies, but numbers were not further increased in patients suffering from COPD [28]. Along the same line, increased ROR*γ*t expression in lung tissue was found in COPD patients and normal smokers compared to healthy control nonsmokers [39]. However, these authors also observed that increased ROR*γ*t levels paralleled the aggravation of the disease [39]. Smoking therefore certainly seems to upregulate IL-17A production in the airways, but it remains unclear whether COPD is associated with further increase in airway IL-17A expression. Differences in microbiota might furthermore be responsible for differences in airway IL-17A cytokine expression [40]. In a recent study, the authors demonstrated that some smokers had less diverse lung microbiota relative to smokers with normal lung function, indicating that alteration in lung microbiota can occur in subjects with no spirometric evidence of disease COPD, but this could be the first signature of disease progression. Although more study is warranted, at least, the results of the cited study provide a framework for characterizing the role of two important pathogens, *Pseudomonas* and *Haemophilus*, in the development, progression, and/or exacerbation of COPD [40]. The role of these two pathogens is certainly well studied in CF patients.

## 4. IL-17A in Cystic Fibrosis

**Figure 1:**
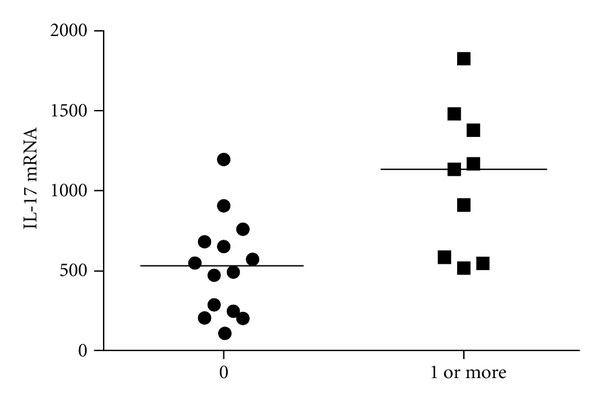
Sputum IL-17A mRNA levels in children with CF. Sputum mRNA was extracted from 23 children with CF aged 5–17 years, as described [47]. IL-17A mRNA levels were measured by real-time RT-PCR [44]. Sputum IL-17A mRNA levels in 9 CF children experiencing at least one exacerbation in the past 12 months (1 or more) were compared to those without exacerbation (*n* = 14). *P < 0.05 by Mann-Withney *U* test.

The major cause of morbidity and mortality in CF is lung damage characterised by bronchiectasis. This damage is the result of the vicious cycle of chronic infection and inflammation with production of harmful products, such as proteases and oxidants, secreted mainly by neutrophils. A major factor in the respiratory health of CF subjects is chronic *Pseudomonas aeruginosa* infection which is associated with a poor clinical outcome [41]. Chronic colonization with *Staphylococcus aureus* and/or *Haemophilus influenzae *also occur but seems to be more frequent in children suffering from CF compared to adults with CF [42]. McAllister and colleagues found elevated IL-17A (and IL-17A-inducing IL-23) protein levels in BAL-fluid and sputum of 8 CF patients during exacerbation [43]. We were the first to show increased sputum IL-17A (and IL-23 mRNA) levels in a group of stable adult CF patients when compared to healthy controls [44]. Chronic infection with *P. aeruginosa *(but not* Staphylococcus aureus) *was associated with higher mRNA expression of these cytokines in CF airways [44]. These results suggested a potential role for the IL-23/IL-17A axis in CF lung inflammation (and/or host response to *P. aeruginosa*) and suggested that the adaptive immune system might be involved in the pathophysiology of CF lung disease. However, the study did not reveal the source of this IL-17A protein expression. Recent data clearly demonstrated the presence of CD4^+^IL-17A^+^ adaptive T cells in airway walls of established CF as well as newly diagnosed CF patients [15]. On the other hand, these authors as well as Brodlie and their coworkers demonstrated that neutrophils might be a second source of IL-17A in CF patients, leading to a potential vicious cycle of neutrophilic inflammation in patients with CF [15, 45]. Consistent with this potential vicious cycle of IL-17A production after initial neutrophil recruitment to the airways, we observed higher sputum IL-17A mRNA levels in children with CF who had at least one exacerbation in the past 12 months, compared with those without exacerbation (Figure 1). We hypothesize that with each exacerbation neutrophils are attracted to the airways upon IL-8 production induced by local IL-17A and they have the potential to secrete IL-17A, leading to chronic neutrophil recruitment, explaining both the increased sputum neutrophil levels and the increased IL-17A levels in patients with stable CF. Moreover, recent data demonstrate that neutrophils from CF patients have deficient bacterial clearance capacity and slower rate of apoptosis compared to healthy subjects [46]. Neutrophils therefore remain in the airways of CF patients, while deficient in bacterial clearance. This in turn might lead to shift in the bacterial expression of virulence factors [46]. 

**Figure 2:**
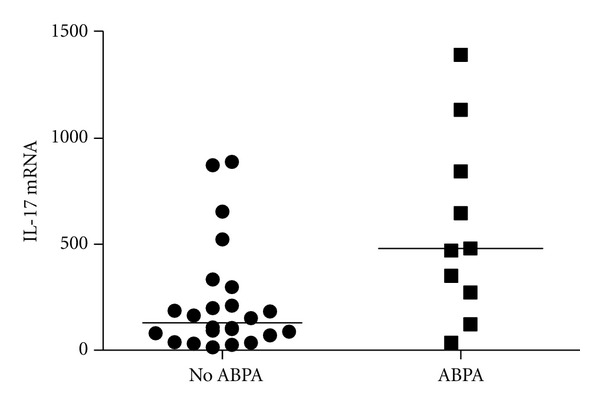
Sputum IL-17A mRNA levels in CF patients without and with ABPA. Sputum mRNA was extracted from adult CF patients without (*n* = 22) and with (*n* = 9) ABPA and IL-17A mRNA levels were measured by real-time RT-PCR [44]. ABPA was defined on the basis of (1) deteriorating lung function, (2) immediate Aspergillus fumigatus (*af*) skin test reactivity, (3) serum total IgE > 1000 IU/mL, (4) elevated *af* specific IgE and IgG antibodies, and (5) chest radiographic infiltrates. *P < 0.05 by Mann-Withney *U* test.

Furthermore, the chronic IL-17A expression might in turn instruct the local microbiota and lead to increased virulence of some pathogenic strains. *Aspergillus fumigatus* can increase its biofilm formation upon binding IL-17A and its filamentation can be promoted, inducing resistance to local antifungal defenses [10]. This might be of utmost relevance in a subgroup of CF patients, namely patients with allergic bronchopulmonary aspergillosis (ABPA). As indicated in Figure 2, and fitting with this hypothesis, we found increased sputum IL-17A mRNA levels in CF patients with APBA, compared to CF patients without ABPA. In these patients, the increased IL-17A levels might, although necessary for the defence against these fungi, in turn be responsible for the chronic aspergillosis. Amongst patients with ABPA, those patients receiving active antifungal treatment and corticosteroids (active APBA) had the highest sputum IL-17A mRNA levels (our own unpublished results).

## 5. IL-17A in Acute Rejection and/or BOS after Lung Transplantation

Lung transplantation is now a well-established treatment option for patients with end-stage pulmonary disease, end-stage cystic fibrosis in particular. Fifty to seventy percent of transplant patients experience one or more episodes of acute rejection within the first three months after transplantation [48, 49]. However, this acute rejection is associated with increased risk of chronic rejection or bronchiolitis obliterans syndrome (BOS), which causes transplant loss and bad clinical outcome [50]. In collaboration with our colleagues from the lung transplantation unit at our university, we were able to demonstrate that IL-17A protein is temporary upregulated in BAL fluid during acute rejection, associated with increased lymphocyte numbers as well as with increased neutrophil numbers (which correlated significantly with the IL-8 protein levels in BAL) [51]. Later on, we also showed that BOS patients have the highest BAL IL-17A (and IL-23) mRNA levels when compared to stable transplant patients and patients with acute rejection; levels were comparable to levels in infected patients [52]. Colonization with gram negatives, fungi, or multiple pathogens was on the contrary, only slightly more present in BOS (9, 2, and 1 (of whom 10 *Pseudomonas*+) on 36, resp.) than in stable transplant (6, 1, and 4 on 42, resp.) patients [52]. These data highlighted the involvement of the IL-23/IL17A axis in lung transplantation rejection. However, the source of the IL-17A production was not studied. Recently, Hodge and colleagues observed that IL-17^+^ NKT-like cells are increased in peripheral blood from lung transplant recipients compared to healthy control subjects [53]. Whether these cells are indeed also responsible for the local increase in IL-17A in BAL fluid from lung transplant patients is not known. Burlingham and colleagues suggested that while alloimmunity initiates lung transplant rejection, *de novo* autoimmunity mediated by collagen V (col(V))-specific Th17 cells as well as monocyte/macrophage accessory cells ultimately causes progressive airway obliteration [54]. Recent data also suggested that a shift in immunodominance of self-antigenic determinants of Col-V results in the induction of IFN-*γ* and IL-17A with loss of tolerance leading to autoimmunity to Col-V, which leads to chronic lung allograft rejection [55].

The impact of IL-17-producing cells in certain respiratory diseases leaded to booming research for new treatment options in those diseases.

## 6. Therapeutic Implications

Inhaled glucocorticosteroids, cornerstone in reducing airway eosinophils and gold standard in asthma treatment, are unfortunately less effective in attacking IL-17-dominated diseases. Glucocorticosteroids are successfully used in some patients with CF (mainly in patients with CF asthma) in whom they might reduce airway eosinophils. Fitting with this hypothesis, we observed a significantly higher sputum eosinophil percentage in CF patients with CF asthma (*n* = 13, mean age 24 years  ±2.6), compared to patients without CF asthma (*n* = 21, mean age 27.1 years  ±6.9) (median 1% versus 0.1%, *P* = 0.03). Asthma was defined in these CF patients based on airway FEV_1_% reversibility and the presence of symptoms: wheezing episodes and/or bronchial hyperreactivity (our own unpublished data). Almost 80% of these CF-asthmatics were daily treated with inhaled corticosteroids and reported clinical benefit of this treatment, whereas only 30% of the patients with CF without asthma used daily ICS treatment (unpublished data). 

On the other hand, severe asthmatics (with neutrophil predominance), COPD patients, CF patients without asthma, and patients with acute rejection and/or BOS after lung transplantation are waiting for additional treatment options. Due to the IL-17 predominance in some of these disease phenotypes, researchers focused on the possibility to reduce local IL-17 production.

 Indirectly, macrolides, and more specifically azithromycin, have been suggested to have anti-inflammatory capacities, besides their anti-infectious potentials, and their effects have been studied in several clinical trials. A recent study suggested no clinical benefit of 12-week azithromycin treatment (weekly dosed) compared to placebo in patients with asthma, although asthmatic patients who received the medication in open label reported improvement of quality of life, asthma symptoms, and asthma control [56]. This could indicate a placebo effect in the open-label-treated group. However, patients volunteered to take part in the placebo-controlled trial and received the active product if they refused to take part in the placebo-controlled trial. This could have induced selection bias towards more severe asthmatics in the open-label arm. These patients probably are more neutrophilic and might therefore be optimal candidates for azithromycin treatment. Open label patients had significantly more chronic sinusitis and less allergy than patients in the randomized trial, fitting with the hypothesis of selection bias [56]. The results of an earlier reported retrospective study also suggested that neomacrolides might be useful as an add-on therapy in patients with severe asthma and/or bronchiectasis. The treatment might be especially useful in older asthmatics with severe (most likely neutrophilic) asthma and/or children with neutrophilic asthma [57, 58]. While awaiting large prospective trials in severe asthmatics, older studies suggested no benefit in young asthmatics, known to be mostly allergic and/or eosinophilic asthmatics if persistent until adulthood [59] and also fitting with the hypothesis that preferentially older asthmatics might benefit from this therapy [60]. 

Azithromycin might offer new options to treat COPD patients [61]. Standard treatment should include smoking cessation, enrollment in a pulmonary rehabilitation program, and the use of evidence-based medications, including long-acting inhaled beta-agonists, long-acting inhaled anticholinergic agents, and inhaled glucocorticoids. Compliant patients with more than two exacerbations in the previous year can be treated with azithromycin [62]. Treatment should be for one year, after which a serious evaluation of benefit should be done, before continuing the treatment later on [62]. Resistance of certain pathogens due to chronic azithromycin use should certainly be studied.

Azithromycin treatment was also studied in CF patients. Since 2002, several studies showed improved clinical outcome of CF patients upon treatment with azithromycin [63–65]. Whether the effect of the treatment is primarily anti-infectious or primarily anti-inflammatory in patients with CF is difficult to disentangle. Long-term, low-dose treatment with azithromycin in CF patients certainly leads to reduced prevalence of *S. aureus*, *S. pneumoniae*, and *H. influenzae*, but might increase macrolide resistance in *S. aureus* in these patients [65]. In a prospective study approved by our local ethical committee, we prospectively recruited 13 adult CF patients (>16 years) in the outpatient clinic of the University Hospitals Gasthuisberg, after obtaining informed consent in 2011. Patients received oral azithromycin (Zitromax, Pfizer, Elsene, Belgium) (250 mg, 3x/week) during 3 months. Lung function parameters and sputum induction were performed as described [44], before and after three months of azithromycin treatment. Paired FEV_1_% predicted (*P* = 0.42) and FVC in liters (*P* = 0.86) remained unaltered (our own unpublished data). Sputum neutrophil percentages and sputum IL-17A mRNA levels also remained stable (our own unpublished data). However, sputum macrophage percentages (median 6% before treatment versus median 3% after treatment, *P* = 0.08) and sputum TNF mRNA levels (median TNF/*β* actin 200 ∗ 10^4^ before treatment versus median TNF/*β* actin 160 ∗ 10^4^ after treatment, *P* = 0.07) tended to decrease after azithromycin treatment (our own unpublished results). These data are in line with the data recently published by Saint-Criq and coworkers showing no effect of azithromycin on *in vitro* IL-8 production by epithelial cell lines, either from cystic fibrosis patients or from healthy subjects [66]. These data are however also in line with the reported ±30% reduction of *in vitro *TNF-alpha mRNA and protein levels produced by cystic fibrosis airway epithelial cell lines treated with azithromycin [67]. Along the same line, TNF production by monocytic THP-1 cells stimulated by lps as well as by murine M1 alveolar macrophages from CF and wild type mice, was also reduced upon *in vitro* treatment with azithromycin [68, 69]. The positive effect of azithromycin in CF patients could be the elegant combination of the anti-infectious and anti-inflammatory properties of the drug, which perhaps could also be attributed to other members of the macrolide family, reducing the risk to develop resistance.

The observation that azithromycin could potentially reduce BOS by reducing neutrophilia as well as IL-8 production [70] is sustained by *in vitro *work, showing inhibition of IL-17-induced IL-8 release from human airway smooth muscle cells when treated with azithromycin [71]. Further study should now further unravel at what moment azithromycin treatment should best be started in lung transplant patients. 

The anti-inflammatory properties of the drug in the cited respiratory diseases could furthermore be related to their potential to improve the phagocytic (and efferocytic) function of lung macrophages [72], which are reported to be defective in some of the cited diseases, such as in COPD [73] and BOS [74]. Several studies suggested that azithromycin could change the macrophage to a more M2 phenotype, which are characterized by specific markers and have increased IL-10 production [75–77]. If its anti-inflammatory effect would mainly be due to the induction of IL-10 gene expression and/or expression and release of this cytokine and specific membrane markers, one could try to develop a component of azithromycin/macrolides with similar function but without its anti-infectious properties. This would reduce its risk of bacterial resistance.

In all the cited diseases, we are awaiting the results of large clinical trials with anti-IL-17A or anti-IL-17R treatment, which will be available in the near future. These biologicals exert their function independent of the source of the local IL-17A overproduction in respiratory IL-17-mediated diseases. Innate or adaptive IL-17? Most likely both pathways are important, but depending on the respiratory disease type, the innate or the adaptive immunity might have a different weight.
